# MHC-I and PD-L1 Expression is Associated with Decreased Tumor Outgrowth and is Radiotherapy-inducible in the Murine Head and Neck Squamous Cell Carcinoma Model MOC1

**DOI:** 10.1007/s11307-024-01934-w

**Published:** 2024-07-15

**Authors:** Daan F. Boreel, Gerwin G. W. Sandker, Marleen Ansems, Renske J. E. van den Bijgaart, Johannes P. W. Peters, Paul N. Span, Gosse J. Adema, Sandra Heskamp, Johan Bussink

**Affiliations:** 1 Radiotherapy and OncoImmunology Laboratory, Department of Radiation Oncology, Radboudumc, Geert Grooteplein Zuid 32, 6525GA Nijmegen, The Netherlands; 2 Department of Medical Imaging, Radboudumc, Geert Grooteplein 10, Nijmegen, 6525GA The Netherlands

**Keywords:** Radiotherapy, Immunotherapy, HNSCC, Tumor microenvironment

## Abstract

**Introduction:**

Combined radiotherapy and immune checkpoint inhibition is a potential treatment option for head and neck squamous cell carcinoma (HNSCC). Immunocompetent mouse models can help to successfully develop radio- immunotherapy combinations and to increase our understanding of the effects of radiotherapy on the tumor microenvironment for future clinical translation. Therefore, the aim of this study was to develop a homogeneous, reproducible HNSCC model originating from the Mouse Oral Cancer 1 (MOC1) HNSCC cell line, and to explore the radiotherapy-induced changes in its tumor microenvironment, using flow cytometry and PD-L1 microSPECT/CT imaging.

**Materials and Methods:**

*In vivo* growing tumors originating from the parental MOC1 line were used to generate single cell derived clones. These clones were screened *in vitro* for their ability to induce programmed cell death ligand 1 (PD-L1) and major histocompatibility complex class I (MHC-I) following IFNγ exposure. Clones with different IFNγ sensitivity were inoculated in C57BL/6 mice and assessed for tumor outgrowth. The composition of the tumor microenvironment of a stably growing (non)irradiated MOC1-derived clone was assessed by immunohistochemistry, flow cytometry and PD-L1 microSPECT/CT.

**Results:**

Low *in vitro* inducibility of MHC-I and PD-L1 by IFNγ was associated with increased tumor outgrowth of MOC1 clones *in vivo*. Flow cytometry analysis of cells derived from a stable *in vivo* growing MOC1 clone MOC1.3D5^low^ showed expression of MHC-I and PD-L1 on several cell populations within the tumor. Upon irradiation, MHC-I and PD-L1 increased on leukocytes (CD45.2^+^) and cancer associated fibroblasts (CD45.2^−^/EpCAM^−^/CD90.1^+^). Furthermore, PD-L1 microSPECT/CT showed increased tumor uptake of radiolabeled PD-L1 antibodies with a heterogeneous spatial distribution of the radio signal, which co-localized with PD-L1^+^ and CD45.2^+^ areas.

**Discussion:**

PD-L1 and MHC-I inducibility by IFNγ *in vitro* is associated with tumor outgrowth of MOC1 clones *in vivo*. In tumors originating from a stably growing MOC1-derived clone, expression of these immune-related markers was induced by irradiation shown by flow cytometry on several cell populations within the tumor microenvironment such as immune cells and cancer associated fibroblasts. PD-L1 microSPECT/CT showed increased tumor uptake following radiotherapy, and autoradiography showed correlation of uptake with areas that are heavily infiltrated by immune cells. Knowledge of radiotherapy-induced effects on the tumor microenvironment in this model can help optimize timing and dosage for radio- immunotherapy combination strategies in future research.

**Supplementary Information:**

The online version contains supplementary material available at 10.1007/s11307-024-01934-w.

## Introduction

Head and neck cancer is the seventh most common cancer worldwide of which head and neck squamous cell carcinoma (HNSCC) comprises the majority of patients [1]. HNSCC includes cancers of the larynx, pharynx, and oral cavity [2]. Most patients are diagnosed at late stages, presenting with locally advanced disease, and have a poor prognosis [1]. Combining radiotherapy with immune checkpoint inhibition holds potential as a curative treatment option due to the anti-tumor immune-enhancing effects of irradiation [3]. However, the combination of radiotherapy with inhibition of programmed cell death protein (PD-1) or its ligand 1 (PD-L1) has not proven to be effective yet for HNSCC patients and several trials are currently ongoing [4–8]. Possible mechanisms contributing to the ineffectiveness of current radio- immunotherapy approaches in HNSCC might be related to dampening of the immune response by elective nodal irradiation or therapy timing [9, 10].

Preclinical studies have indicated that radiotherapy-immunotherapy combinations may be more effective than monotherapies [11–14]. Other studies suggest that additional modulation of the tumor microenvironment (TME) might be necessary to achieve the full potential of these combined treatments [15–19]. To better understand the potential synergistic effects of radiotherapy and immune checkpoint inhibition, in-depth mechanistic studies in preclinical syngeneic HNSCC tumor models are needed. For instance, the evolving TME -including immune cells and cancer associated fibroblasts (CAFs)- is known to play an important role in tumor growth and progression [19–21]. Moreover, expression of immune-related markers upregulated by Interferon gamma (IFNγ) signaling like PD-L1 and major histocompatibility complex class I (MHC-I) in the TME, i.e. on non-tumor cells, have been linked to improved prognosis and reduced tumor outgrowth, although some contradictory reports exist [22–27]. Understanding of immune response-related markers and the radiation-induced effects on them are necessary to determine the optimal timing, response prediction, and/or sensitization to radio- immunotherapy combinations in HNSCC.

Only a limited number of murine HNSCC models are available and the most well-studied one is murine oral cancer 1 (MOC1), which has been used before to demonstrate the efficacy of radio- immunotherapy combination strategies [11, 28, 29].

In this study, we aim to develop a homogeneous, reproducible HNSCC model of MOC1 origin after *in vivo* passage of the parental MOC1 cells, selected based on IFNγ-inducibility of immunological markers MHC-I and PD-L1. Furthermore, we aim to explore radiotherapy-induced changes in the TME of this tumor model. Using single-cell-derived clones, we showed that *in vitro* MHC-I and PD-L1 expression was associated with *in vivo* tumor outgrowth. Additionally, in the selected MOC1 clone, radiotherapy-induced expression of MHC-I on leukocytes and PD-L1 on leukocytes and CAFs *in vivo*. By PD-L1 microSPECT/CT, an increased tumor uptake was measured following radiotherapy. This knowledge will contribute to optimizing the efficacy of radio- immunotherapy combinations in HNSCC.

## Material and Methods

### Cell Lines

MOC1 (provided by R. Uppaluri, Dana-Farber Cancer Institute, Boston) cells or single cell-derived MOC1 clones were cultured in 2:3 Iscove’s Modified Dulbeco’s Medium, 1:3 Ham’s F-12 nutrient mix with 5% Fetal Calf Serum, 1% penicillin–streptomycin (all Gibco), 5 ng/mL Epidermal Growth Factor (EMD Millipore), 40 ng/mL hydrocortisone and 5 μg/mL insulin (both Sigma Aldrich) at 37 °C in 5% CO_2_ and 20% O_2._ Cells were stimulated with recombinant murine IFNγ (5 ng/mL) (Peprotech) for 24 h. For *in vitro* experiments, cells were not passaged more than fifteen times post thawing. For *in vivo* experiments, cells were passaged two times post thawing.

### Mice

All animal experiments were conducted in accordance with the principles laid out by the Dutch Act on Animal Experiments (2014) and approved by the Animal Welfare Body of the Radboud University Nijmegen and Central Authority for Scientific Procedures on Animals. In all animal experiments, female C57BL/6 mice that can be housed together in groups (10–12 weeks, Charles River) were used. Mice were housed in individually ventilated cages with filter top (IVC Blueline, Tecniplast) and could eat and drink ad libitum. In all experiments animals were assigned to groups by block randomization based on tumor volume. Tumor size was determined by caliper measurement by biotechnicians that were blinded to experimental groups. Tumor volume was calculated using the following formula:

$$Tumor\;volume=Height\times{Width}^2\times0.5$$

### Fluorescence-Activated Cell Sorting of *In Vivo* Passaged Tumor Cells

Mice were subcutaneously (s.c.) inoculated with 0.5 × 10^6^ or 1.0 × 10^6^ MOC1 cells in phosphate buffered saline (PBS) on the right hindleg to monitor tumor growth (n = 3 per group). At day 57 post injection mice were sacrificed and tumors were excised, mechanically dissociated in Serum Free RPMI (Gibco) with 1 mg/mL collagenase type III (Worthington) and 30 μg/mL DNase type I (Roche), and incubated for 30 min at 37 °C. EDTA was added to a final concentration of 1 mM and cells were passaged twice over an 100 µm cell strainer (BD Falcon) to retrieve a single cell suspension. 1.0 × 10^6^ cells were seeded in a T75 culture flask (Corning) and expanded. For fluorescence-activated cell sorting cells were harvested and incubated for 15 min at 4 °C with rat anti-EpCAM-FITC (1:400, eBioscience). EpCAM-positive cells were sorted as single cells using a FACSAria II SORP sorter (BD Bioscience) and further expanded *in vitro*.

### *In vitro* Radiosensitivity and Cell Growth

To assess radiosensitivity, colony-forming assays were performed as previously described using a 320 kV Xrad irradiator (RPS Services Limited) at a dose rate of 3.8 Gy/min [30]. *In vitro* cell growth was measured using the IncuCyte ZOOM Live-Cell Analysis System (Essen BioScience). For MOC1 parental or MOC1 clones, 500 cells/well were seeded in 96-wells plates (Corning). Images were retrieved and confluency was calculated as previously described [31]. No differences in cell viability were observed. Doubling time was calculated using the following formula:

$$Doubling\;time=\frac{Time\times\log(2)}{(\log(Final\;confluency)-\log\;(Initial\;confluency)}$$

### Comparison of MOC1 Clone’s Outgrowth

MOC1 clones were selected based on the inducibility by IFNγ of MHC-I and PD-L1; 3D5 (MOC1.3D5^low^), 4E8 (MOC1.4E8^intermediate^), 4B9 (MOC1.4B9^high^). To compare tumor growth of MOC1 clones, mice were inoculated s.c. with 0.5 × 10^6^ cells of MOC1.3D5^low^, MOC1.4E8^intermediate^, MOC1.4B9^high^ and MOC1 parental in 1:3 matrigel (BD Bioscience): PBS on the right hindleg (n = 5 per group). Mice were sacrificed when tumors reached a size of approximately 400 mm^3^. Half of the tumor was dissociated for flow cytometry analysis as described above, and half of the tumor was snap-frozen in liquid nitrogen for immunohistochemistry (IHC) analyses.

### Antibodies and Flow Cytometry Analysis

Single cell suspension harvested from *in vitro* cultured cells, or single cell suspension of *in vivo* grown tumors were used. Cells were incubated with viability dye eFluor™ 780 or eFluor™ 450 (eBioscience) in PBS for 15 min at 4 °C to distinguish living and dead cells. Before staining cells were washed twice in 0.5% serum albumin and 0.05% sodium azide in PBS (PBA) and incubated with anti-CD16/CD32 Fc-block (BD) for 15 min at 4 °C. Cells were washed twice in PBA and stained for 30 min at 4 °C using the following antibodies or appropriate isotype controls: CD90.2-BV510 (Thy-1.2), MHC-I-PE (H-2 Kb/H-2D^b^), CD45.2-A647 (Ly5.2), Ly6G-BV605, CD3-BV510, CD45.2-PerCP/Cy5.5 (Ly5.2), IgG2b, k-BV510, IgG2a, k-PE, IgG2a, k-PE/Cy7, IgG2a, k-APC from Biolegend, PD-L1-APC (CD274), CD11c-BUV395 (ITGAX), CD11b-BUV737 (ITGAM), Ly6C-A700 from BD Bioscience, EpCAM-FITC (CD326), PD-L1-PE/Cy7 (CD274), CD25-APC (IL2RA), FoxP3-PE/Cy7 from eBioscience and CD45.2-FITC (Ly5.2), F4/80-PE/Cy7 (EMR1), CD4-APC/Cy7, CD8β.2-FITC (Ly-3), NK1.1-PE from Antibodychain. After staining cells were washed in PBA and measured on the FACSCantoII (BD) or CytoFLEX LX Flow Cytometer (Beckman Coulter). Data were analyzed using FlowJo V10.7 (Tree Star) as previously described [32].

### Irradiation of Mice for Flow Cytometry Analysis

For further characterization we selected clone MOC1.3D5^low^, which consistently grew out into solid tumors. To determine effects of irradiation on the TME, MOC1.3D5^low^ cells were inoculated on the right hindleg and when tumors reached a size of 200 mm^3^ were irradiated with a single dose of 6, 10 or 18 Gy using a 320 kV Xrad irradiator at a dose rate of 3.8 Gy/min or received no irradiation. To ensure irradiation of only the tumor-bearing right hindleg the rest of the mice body was shielded by lead. Tumors were harvested at 3 (n = 6 per group) and 10 (n = 3 per group) days post irradiation and dissociated for flow cytometry analysis as described above.

### PD-L1 microSPECT/CT Imaging of (Non-)irradiated Mice

MOC1.3D5^low^ cells were inoculated on the right hindleg and when tumors reached a size of 200 mm^3^, tumors were irradiated with a single dose of 10 Gy using a 320 kV Xrad irradiator at a dose rate of 3.8 Gy/min ([^111^In]In–anti–mPD-L1 group), with a clinical 10 MV photon beam at a dose rate of 6 Gy/min (Elekta) ([^111^In]In-IgG2b group) or received no irradiation (n = 6 per group). Rat anti-mPD-L1 (clone 10F.9G2, Bio X Cell) or rat IgG2b (clone LTF-2, Bio X Cell) was labeled with Indium-111 (^111^In) as previously described [33, 34]. 24 h post irradiation mice received 30 μg [^111^In]In–anti–mPD-L1 (0.48 MBq/µg) or 30 μg [^111^In]In-IgG2b (0.53 MBq/µg) intravenously in the tail vein. 24 h-post tracer administration SPECT/CT images were acquired with the U-SPECT-II/CT system (MILabs). Mice were scanned using the following acquisition settings: 30 min acquisition time, 1.0 mm-diameter pinhole mouse high sensitivity collimator, and CT parameters of 160-µm spatial resolution, 615 µA, and 65 kV. Data were reconstructed using the MILabs software (version 2.04) using the following settings: energy windows at 171 keV (range 154 to 188 keV) and 245 keV (range 220 to 270 keV), 1 iteration, 16 subsets, and a 0.4 mm voxel size. Maximum intensity projections were created using VivoQuant. After SPECT/CT imaging mice were dissected, tumor and selected organs were weighed and radioactivity was measured using a γ-counter (2480 Wizard, Perkin-Elmer). Results are presented as percentage injected dose per gram tissue (%ID/g). Tumors were snap-frozen in liquid nitrogen for IHC analyses.

### Immunohistochemistry and Autoradiography

For IHC analyses, frozen tumors were sectioned (5 μm), mounted on poly-l-lysine coated slides and fixed in ice-cold acetone for 10 min. For the IHC staining, sections were blocked with 5% normal donkey serum (30 min, RT) (Jackson ImmunoResearch) and incubated (30 min, 37 °C) with rat anti-MHC-I (1:200, Invitrogen), goat anti-PD-L1 (1:100, Biotechne), biotinylated rat anti-EpCAM (1:200, Invitrogen), biotinylated mouse anti-CD45.2 (1:100, Biolegend), biotinylated rat anti-MHC-II (1:200, Antibodychain) or 9F1 against mouse vessels (Radboudumc). Subsequently, secondary antibodies were applied (45 min, 37 °C); for MHC-I chicken anti-rat Alexa647 (1:100, Invitrogen), for PD-L1 donkey anti-goat F(ab’)_2_ fragment Cy3 (1:300, Jackson Immunoresearch) or donkey anti-goat Alexa488 (1:600, Invitrogen), for EpCAM chicken anti-rat Alexa647 (1:100), for CD45.2 donkey anti-mouse F(ab’)_2_ fragment Alexa488 (1:100, Jackson ImmunoResearch) or mouse anti-biotin Cy3 (1:100, Jackson ImmunoResearch), for MHC-II mouse anti-biotin Cy3 (1:100) and for 9F1 chicken anti-rat Alexa647 (1:100). Whole-tissue section gray-scale images (pixel size, 2.59 × 2.59 μm) MHC-I, PD-L1, EpCAM and CD45.2 were obtained as previously described [35]. For Autoradiography (AR), frozen tumor sections (5 μm) of mice injected with [^111^In]In–anti–mPD-L1 were mounted on poly-l-lysine coated slides and exposed to a Fujifilm BAS 2025 photosensitive plate (Fuji Photo Film). Plates were scanned using an AS-1800 II bioimaging analyzer at a pixel size of 25 × 25 μm. AR images were analyzed using Aida Image Analyzer software (Raytest). Spatial correlation between IHC and AR images was assessed as previously described [36].

### Statistical Analysis

Statistical analyses were performed using GraphPad Prism (version 8.0). Results are expressed as mean ± SD unless indicated otherwise. Unpaired t-test was used to compare 2 groups. One-way ANOVA was used to compare > 2 groups. A chi-squared test was used to compare tumor outgrowth between groups. A spearman test was used to assess correlations between values. p values of 0.05 or less were considered significant.

## Results

### Characterization of Single Cell-derived MOC1 Clones

**Fig. 1:**
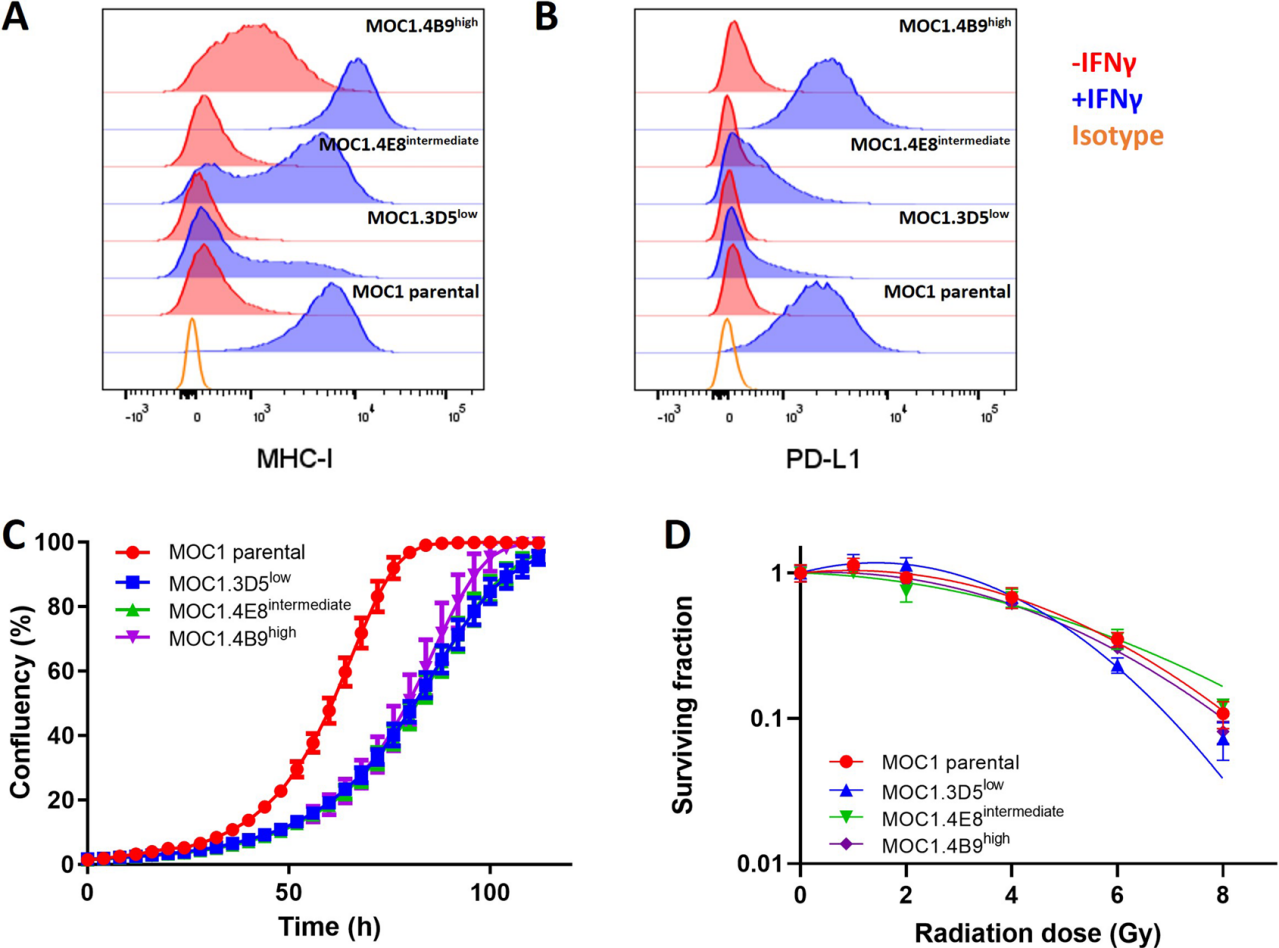
*In vitro* characterization of MOC1 parental cells and MOC1 clones. (**A**) MHC-I expression and (**B**) PD-L1 expression of MOC1 parental, MOC1.4B9^high^, MOC1.4E8^intermediate^ and MOC1.3D5^low^, unstimulated or treated with IFNγ (5 ng/mL, 24 h). Typical example of 3 independent experiments. (**C**) *In vitro* growth curves of MOC1 parental, MOC1.4B9^high^, MOC1.4E8^intermediate^ and MOC1.3D5^low^. (**D**) *In vitro* radiosensitivity of MOC1 parental, MOC1.4B9^high^, MOC1.4E8^intermediate^ and MOC1.3D5^low^. Graph C and D show mean ± SD

MOC1 parental cells inoculated on the right hindleg of C57BL/6 mice showed heterogenous tumor outgrowth *in vivo,* irrespective of the implantation protocols used (i.e. tumor cell suspension in PBS or Matrigel) (Supplementary Fig. S1). To develop a syngeneic mouse model with a more predictable tumor growth, a MOC1 tumor was dissociated into single cells and re-plated to obtain MOC1 clones with reproducible and homogeneous tumor growth *in vivo*. Hereto, the tumor cells were single cell-sorted by their expression of epithelial cell adhesion molecule (EpCAM). Subsequent flow cytometry analysis of 44 different MOC1 clones plus the MOC1 parental cell line revealed a great heterogeneity in their IFNγ inducibility of PD-L1 and MHC-I expression (data not shown). Based on these findings, a high-inducible (MOC.4B9^high^), intermediate-inducible (MOC1.4E8^intermediate^) and low-inducible (MOC1.3D5^low^) clone were selected (Fig. 1A-B). Clones that were high-inducible for PD-L1 expression were also high-inducible for MHC-I expression and vice versa. Additional characterization of these MOC1 clones revealed a similar *in vitro* doubling time for all clones (MOC1.3D5^low^ = 15.95 ± 0.45 h, MOC1.4E8^intermediate^ = 15.87 ± 0.36 h, MOC1.4B9^high^ = 15.48 ± 0.25 h), which was significantly longer compared to the MOC1 parental cell line (12.23 ± 0.47 h, p = 0.0001) (Fig. 1C). Importantly, irradiation of MOC1 clones showed no significant differences in the surviving fraction between the MOC1 parental cell line and the clones (Fig. 1D).

### Tumor Outgrowth of Single Cell-derived MOC1 Clones is Associated with MHC-I and PD-L1 Expression and Inducibility

**Fig. 2:**
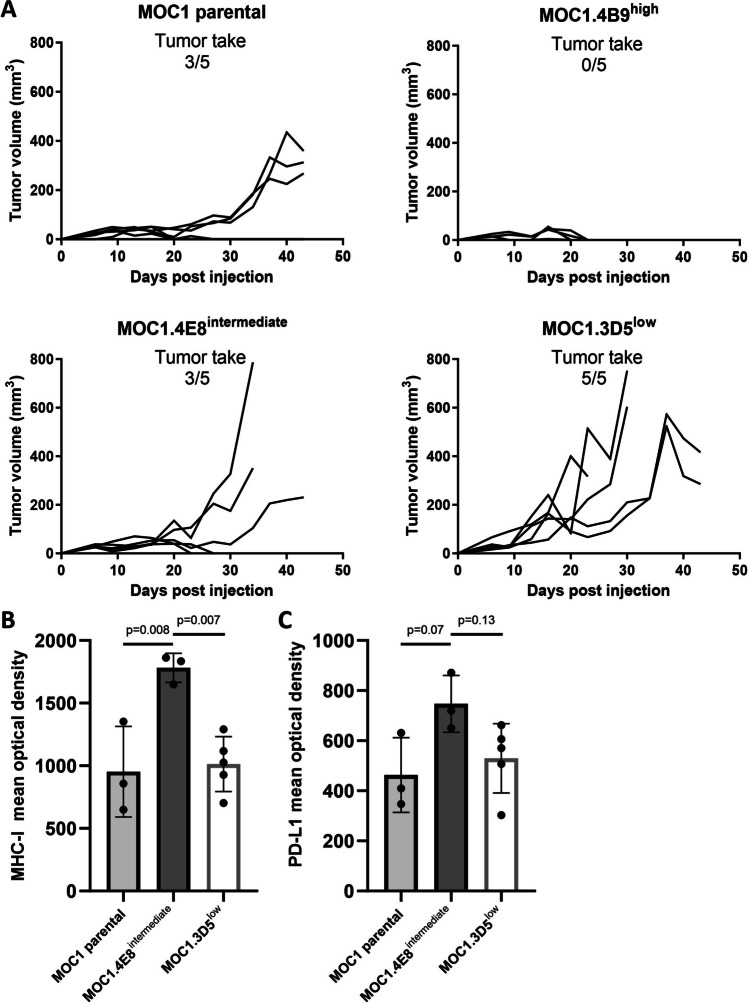
*In vivo* tumor outgrowth is associated with decreased PD-L1 and MHC-I inducibility *in vitro* and expression *in vivo* (**A**) Tumor growth of MOC1 parental, MOC1.4B9^high^, MOC1.4E8^intermediate^, and MOC1.3D5^low^ (**B**) Mean optical density of tumor sections stained for MHC-I. (**C**) Mean optical density of tumor sections stained for PD-L1. All graph bars show mean ± SD

To compare *in vivo* tumor outgrowth of MOC1 clones, mice were inoculated with the MOC1 parental, MOC1.4B9^high^, MOC1.4E8^intermediate^ or MOC1.3D5^low^ cells on the right hindleg. Tumor outgrowth rates were significantly different between the different clones (p = 0.02) (Fig. 2A). We observed no differences in the presence of several subsets of myeloid cells (CD11b^+^, F4/80^+^, CD11c^+^, Ly6G^+^ and Ly6C^+^) and lymphocytes (NK1.1^+^, CD3^+^, CD8^+^, CD4^+^ and Tregs) (Supplementary Fig. S2 and S3) in the TME of the different sub-clones as well as the MOC1 parental cell line. IHC analysis of the tumor tissue for MHC-I and PD-L1 expression showed a significantly increased mean optical density for MHC-I in MOC1.4E8^intermediate^ compared MOC1.3D5^low^ (p = 0.007) and MOC1 parental tumors (p = 0.008) (Fig. 2B and supplementary Fig. S4). A similar trend was observed for the mean optical density of PD-L1 (Fig. 2C). These findings suggest that the lower *in vitro* observed inducibility of MHC-I and PD-L1 of MOC1.3D5^low^ was retained *in vivo* and may have a role in tumor outgrowth.

### Radiotherapy Induces Upregulation of MHC-I and PD-L1 Expression on Several Cell Populations within the Tumor Microenvironment of MOC1.3D5^low^

**Fig. 3:**
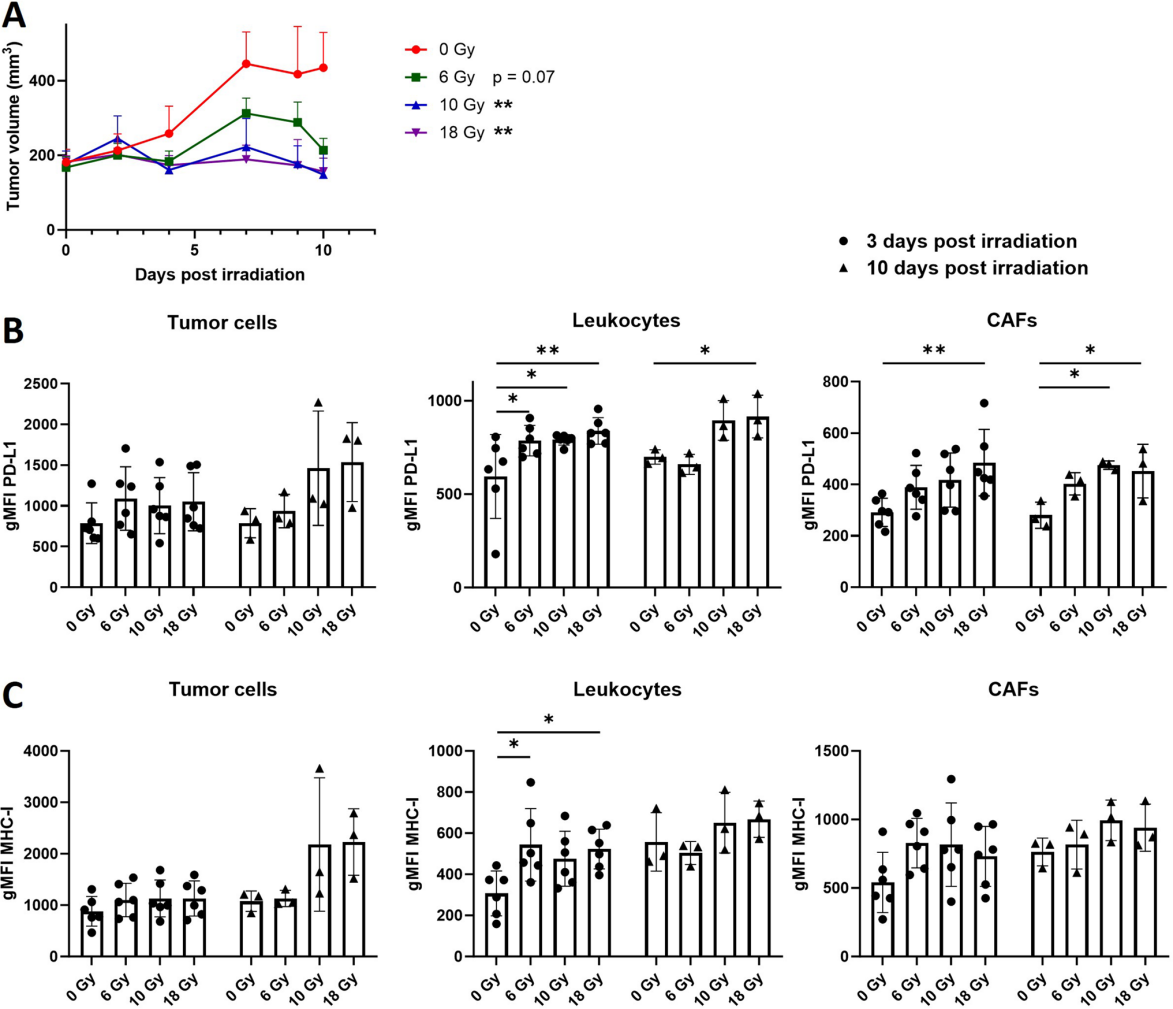
Radiotherapy induces PD-L1 and MHC-I expression on several cell populations within the TME of MOC1.3D5^low^ tumors. (**A**) *In vivo* tumor growth of MOC1.3D5^low^ tumors after irradiation. Graph shows mean ± SEM. (**B**) Geometric mean fluorescent intensity (gMFI) of PD-L1 signal and (**C**) gMFI of MHC-I signal on MOC1.3D5^low^-derived CD45.2^−^/EpCAM^+^ tumor cells, CD45.2^+^ leukocytes and CD45.2^−^/EpCAM^−^/CD90.1^+^ CAFs. Graph bars show mean ± SD

To investigate whether irradiation also influences expression of these factors on tumor cells and other cell populations within the TME, mice bearing stably growing MOC1.3D5^low^ tumors were irradiated with 6, 10 or 18 Gy. The TME was analyzed by flow cytometry at 3 and 10 days post-irradiation to compare early effects with effects occurring at a later stage. The dose range chosen is similar to the biological effective dose of earlier studies investigating fractionated radiotherapy [11]. Tumor volume was significantly reduced in mice irradiated with 10 Gy (p = 0.008) or 18 Gy (p = 0.005) at 10 days post irradiation (Fig. 3A). Flow cytometry analysis of the TME revealed both PD-L1 and MHC-I expression on CD45^+^ leukocytes, CD45.2^−^/EpCAM^+^ tumor cells and CD45.2^−^/EpCAM^−^/CD90.1^+^ CAFs (Fig. 3B-C). Moreover, irradiation of the tumor induced a dose dependent upregulation of PD-L1 on leukocytes and CAFs at 3 and 10 post irradiation, and a significant upregulation of MHC-I expression on leukocytes at 3 days post irradiation (Fig. 3B-C). The percentage PD-L1 and MHC-I-positive cells of the parent populations are provided in Supplementary Figure S5 and showed an additional increase of PD-L1-positive EpCAM^+^ tumor cells at 10 days post irradiation. Increase of PD-L1 and MHC-I gMFI on MOC1.3D5^low^ tumor cells was also observed *in vitro* at 24 h post irradiation, although isotype control staining also slightly increased (Supplementary Fig. S6).

### Radiotherapy Enhances Uptake of [^111^In]In-anti-mPD-L1 in MOC1.3D5^low^ Tumors

**Fig. 4:**
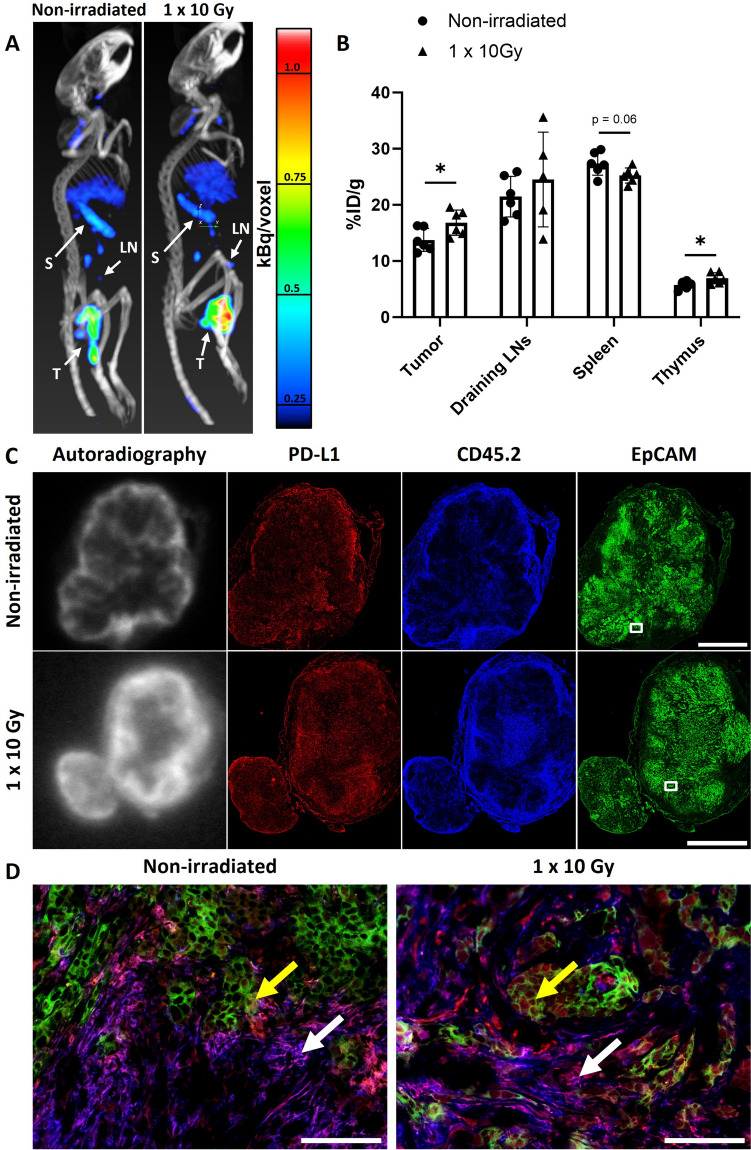
1 × 10 Gy irradiation increases [^111^In]In-anti-mPD-L1 uptake in MOC1.3D5^low^ tumor tissue. (**A**) Maximum intensity projections of [^111^In]In-anti-mPD-L1 microSPECT/CT in mice with non-irradiated and irradiated (1 × 10 Gy) tumors 24 h post tracer injection and 48 h post irradiation. Tumor (T), Spleen (S) and tumor-draining inguinal lymph node (LN) are indicated by white arrows. Scale indicating uptake in kBq/voxel. (**B**) Uptake of [^111^In]In-anti-mPD-L1 in tumor, tumor-draining inguinal lymph node spleen and thymus of mice with non-irradiated and irradiated (1 × 10 Gy) tumors (%ID/g) 24 h post tracer injection and 48 h post irradiation. Graph bars show mean ± SD. (**C**) Autoradiography and IHC staining of PD-L1 (CD274, *red*), leukocytes (CD45.2, *blue*) and EpCAM (CD326, *green*) of the same tumor section of non-irradiated and irradiated (1 × 10 Gy) tumors. Scalebar represents 2.0 mm. (**D**) Color merge images of selected areas of non-irradiated and irradiated (1 × 10 Gy) tumors. PD-L1 (*red*), leukocytes (*blue*), EpCAM (*green*). White arrows indicate PD-L1^+^/CD45.2^+^ areas, yellow arrows indicate PD-L1^+^/EpCAM^+^ areas. White window in C indicates location of image on whole tumor section. 20 × magnification, scalebar represents 100 μm

To investigate how radiatiotherapy affects tumor uptake of PD-L1 antibodies, [^111^In]In-anti-mPD-L1 microSPECT/CT was performed in mice bearing irradiated (10 Gy single dose) or non-irradiated MOC1.3D5^low^ tumors. Tumor, spleen and tumor-draining inguinal lymph nodes could be clearly distinguished on the PD-L1 microSPECT/CT image (Fig. 4A). A microSPECT/CT coronal view is provided in Supplementary Figure S7. Increased intensity of the [^111^In]In-anti-mPD-L1 microSPECT signal was observed in irradiated tumors compared to non-irradiated tumors, which was confirmed by ex vivo quantification of [^111^In]In-anti-mPD-L1 uptake (16.8 ± 2.3 versus 13.8 ± 2.0%ID/g, p = 0.03) (Fig. 4B). No difference in [^111^In]In-anti-mPD-L1 uptake was observed in tumor-draining inguinal lymph node while a trend towards reduced [^111^In]In-anti-mPD-L1 uptake in the spleen following irradiation was observed (p = 0.06). [^111^In]In-anti-mPD-L1 uptake in the thymus was increased following irradiation (p = 0.04). To test if enhanced uptake of [^111^In]In-anti-mPD-L1 could be a result of radiotherapy-enhanced perfusion, increased enhanced permeability and retention effects, or influx of Fc receptor-positive immune cells, we performed a separate biodistribution study using an [^111^In]In-IgG2b isotype control antibody. Irradiation did not increase the tumor uptake of [^111^In]In-IgG2b (Supplementary tab. S1). Although the total tumor uptake of [^111^In]In-IgG2b was higher than that of [^111^In]In-anti-mPD-L1, this can be attributed to the slower blood clearance of [^111^In]In-IgG2b because of the lack of “sink” organs such as the spleen [33, 37]. This is also reflected in the lower tumor-to-blood ratio of [^111^In]In-IgG2b compared with [^111^In]In-anti-mPD-L1. Complete biodistribution data are described in Supplementary Table. S1.

Autoradiography of tumor sections from mice injected with [^111^In]In-anti-mPD-L1 showed a heterogenous [^111^In]In-anti-mPD-L1 distribution, with increased tracer uptake in the tumor rim. IHC analysis shows that the tumor rim mostly consisted of CD45.2^+^ immune cells, both for irradiated and non-irradiated tumors (Fig. 4C). Some PD-L1 expression was observed in EpCAM^+^ tumor areas (Fig. 4D, yellow arrow), however most PD-L1 was present in the CD45.2^+^ compartments of the TME as can be seen in 20 × maginification images (Fig. 4D, white arrow). Moreover, the PD-L1 autoradiography signal correlated with both the CD45.2 signal (non irradiated: *ρ* = 0.38 ± 0.19 irradiated: *ρ* = 0.26 ± 0.19) and the PD-L1 signal (non irradiated: *ρ* = 0.34 ± 0.14 irradiated: *ρ* = 0.35 ± 0.09) as determined by coregistration and quantitative analysis of autoradiography and IHC (Supplementary Fig. S8.). Further IHC analysis revealed co-localization of CD45.2 with MHC-II, suggesting most leukocytes in this tumor are myeloid cells (Supplementary Fig. S9). Together, these findings show that PD-L1 expression is heterogeneous within the TME and, in line with the flow cytometry data, that radiotherapy induces PD-L1 expression in the MOC1.3D5^low^ TME.

## Discussion

Radio- immunotherapy combination strategies are under investigation as a potential treatment option for HNSCC, but have not yet been proven effective [5, 7]. To optimize the efficacy and to steer optimal implementation of radio- immunotherapy into the clinic, in-depth knowledge on the mechanism of action is required. This can be obtained from well-characterized pre-clinical syngeneic mouse tumor models. In this study, we generate three clones derived from the syngeneic mouse oral cancer MOC1 and investigated the influence of immunological markers PD-L1 and MHC-I on tumor outgrowth and investigate the influence of radiotherapy on the TME of MOC1.3D5^low^.

*In vitro* analysis of MOC1-derived clones originating from the same parental cell line reveals a great heterogeneity in the inducibility of PD-L1 and MHC-I expression by IFNγ, while no differences are observed in their *in vitro* growth rate or radiosensitivity. This heterogeneity within a single model may affect processes such as anti-tumor immunity and consequently therapy response as in the real-life situation [26]. Although we characterized separate clones to develop a homogeneous and reproducible model to minimize the variation between experiments and the number of mice per study, it has to be noted that this is not a perfect reflection of the clinical situation where tumor heterogeneity is a general phenomenon.

Interestingly, the *in vitro* IFNγ-inducibility of PD-L1 and MHC-I correlates with tumor outgrowth. Mice inoculated with MOC1.3D5^low^ showed consistent tumor outgrowth, while no tumor outgrowth was observed in MOC1.4B9^high^. These differences in tumor outgrowth might be a result of decreased IFNγ pathway gene expression (e.g. MHC-I and PD-L1), which is a well-known mechanism for immunotherapy resistance and decreased tumor outgrowth [26, 27]. Several other studies showed heterogeneity in tumor growth and therapy efficacy between single cell-derived clones from the same parental cell line that are dependent on tumor cell interactions with the immune-microenvironment [21, 38, 39]. In the tumors that developed from MOC1.4E8^intermediate^, MOC1.3D5^low^ and the MOC1 parental cell line, no difference in infiltration of immune cells is detected, although this could not be investigated in MOC1.4B9^high^ that did not grow out. Based on these observations it is unlikely that differences in immune cell infiltration are the reason for differences in tumor outgrowth. A possible mechanism explaining the consistent outgrowth of clone 3D5^low^ could be the decreased recognition of the tumor cells by the immune system through its expression of MHC-I, as has been suggested in neuroblastoma and triple negative breast cancer [40, 41]. Whether decreased PD-L1 expression has a role in increased tumor outgrowth remains controversial, as PD-L1 is an immunoregulatory pathway [26, 42].

The difference in expression of MHC-I and PD-L1 between MOC1.3D5^low^ and MOC1.4E8^intermediate^ *in vitro* is maintained after transplantation *in vivo* as assessed by IHC. Interestingly, contrary to its *in vitro* inducibility, also the (heterogenous) MOC1 parental cell line shows decreased expression of MHC-I and PD-L1 *in vivo*. This emphasizes that *in vitro* findings are not always translatable to the *in vivo* situation [43]. A potential explanation for this might be *in vivo* selection of the low MHC-I expressing cells that can grow out as they are not recognized by the immune system [44, 45].

*In vitro* irradiation of MOC1.3D5^low^ cells increases the expression of PD-L1 and MHC-I. Flow cytometry analysis of MOC1.3D5^low^ tumors also shows upregulation of PD-L1 on leukocytes (CD45.2^+^) and CAFs (EpCAM^−^/CD45.2^−^/CD90.1^+^) and upregulation of MHC-I on leukocytes after irradiation. As CD90.1 can be expressed on multiple cell types including endothelial cells and pericytes, we cannot exclude that these cell types contribute to the observed PDL1 signal [46, 47]. However, because CAFs make up a significant part of the TME in HNSSC, and CD90.1 is an emerging marker for CAFs, we assume the CD45.2^−^/EpCAM^−^/CD90.1^+^ population to consist mainly of CAFs [48, 49]. A marker that could be used to further characterize CAFs is fibroblast activation protein (FAP) that is also targeted for PET imaging of cancer [50].

[^111^In]In-anti-mPD-L1 microSPECT/CT imaging of MOC1.3D5^low^ tumors shows increased tracer uptake in irradiated compared to non-irradiated tumors. Higher tracer uptake is observed at the tumor rim compared to the core. IHC analysis reveals that this uptake correlates with stroma that is highly infiltrated by CD45.2^+^ leukocytes. Albeit at lower intensity, also EpCAM^+^ tumor areas exhibit PD-L1 expression, revealing PD-L1 expression by different cell populations throughout the TME [51]. The small reduction of [^111^In]In-anti-mPD-L1 uptake in spleen following irradiation was not observed in other tumor models, but could be the result of migration of leukocytes towards the tumor [34]. Our findings show that radiotherapy not only enhances PD-L1 and MHC-I expression on tumor cells, as observed in other studies, but also on several other, non-tumor cell populations within the TME [34, 52]. These radiation-induced changes could potentially serve as a target when radiotherapy is combined with immunotherapy. An example of such a change could be the upregulation of PD-L1 expression on host cells, which is suggested to be essential for immunotherapy efficiency [37, 53]. Moreover, other studies show upregulation of different immune checkpoints like VISTA, TIM-3 and PD-1 in HNSCC and immune cells following irradiation, potentially contributing to immunotherapy efficacy against these targets [54–56]. Imaging of PD-L1 and other immune checkpoints at different time points after radiotherapy, preferably using radiotracers with a short half-life labeled with a positron-emitting radionuclide to enable PET imaging, could potentially show these dynamic expression levels in patients [57]. Further analysis of subsets of immune cells and CAFs, or analysis of different aspects of the environment (e.g. hypoxic areas, tumor stroma, etc.), might reveal different responses to irradiation within these cell populations and environments that might provide a reasoning for immune- radiotherapy combination strategies [36, 58].

The kinetics of radiation-induced changes on the TME could provide a rationale to determine the optimal timing of immunotherapy administration and supports longitudinal monitoring during therapy, for instance by PD-L1 imaging. It is suggested that concurrent radio- immunotherapy, generally applied in most HNSCC trials, might potentially be counter-effective as tissue-resident immune cells are irradiated during the course of treatment and thereby die as a result. We and others show that PD-L1 and MHC-I are upregulated at least up to 10 days post irradiation [11]. Immunotherapy administration after radiotherapy, sparing activated immune cells, might therefore be preferable as has been successful in non-small-cell lung cancer [59].

## Conclusions

In summary, MOC1.3D5^low^ shows consistent and high tumor take and is a reproducible syngeneic mouse model for radiotherapy and immunotherapy research. Stable tumor outgrowth is correlated with decreased inducibility of PD-L1 and MHC-I in MOC1-derived clones. Lastly, PD-L1 and MHC-I is expressed on multiple cell populations within the TME as observed by flow cytometry, and can be upregulated by radiotherapy in several subsets of cells. Moreover, an increased tumor uptake of [^111^In]In-anti-mPD-L1 was seen following radiotherapy. This knowledge can help to optimize radio- immunotherapy combination strategies (e.g. timing) in HNSCC and longitudinal assessment of the TME by means of PD-L1 imaging may serve as a biomarker in clinical translation.

## Supplementary Information

Below is the link to the electronic supplementary material.

Supplementary file1 (DOCX 7.36 MB)

## Data Availability

The data that support the findings of this study are available from the corresponding author upon reasonable request.
