# Supplementary material for: MHC-I and PD-L1 Expression is Associated with Decreased Tumor Outgrowth and is Radiotherapy-inducible in the Murine Head and Neck Squamous Cell Carcinoma Model MOC1
Source: Mol Imaging Biol. 2024 Jul 15;26(5):835–46. doi: 10.1007/s11307-024-01934-w (PMC11436446; doi:10.1007/s11307-024-01934-w)
Supplement: Supplementary file 1 — Supplementary file1 (DOCX 7.36 MB) [file 11307_2024_1934_MOESM1_ESM.docx]

**Electronic supplementary material**

**MHC-I and PD-L1 expression is associated with decreased tumor outgrowth and is radiotherapy-inducible in the murine head and neck squamous cell carcinoma model MOC1**

**Journal: Molecular Imaging and Biology**

Daan F. Boreel^1,2^, Gerwin G.W. Sandker^2^, Marleen Ansems^1^, Renske J.E. van den Bijgaart^1^, Johannes P.W. Peters^1^, Paul N. Span^1^, Gosse J. Adema^1^, Sandra Heskamp^2^, Johan Bussink^1^

^1^Radiotherapy and OncoImmunology laboratory, Department of Radiation Oncology, Radboudumc, Geert Grooteplein zuid 32, 6525GA Nijmegen, The Netherlands

^2^Department of Medical Imaging, Radboudumc, Nijmegen, Geert Grooteplein 10, 6525GA The Netherlands

Corresponding author: Daan F. Boreel, daan.boreel@radboudumc.nl
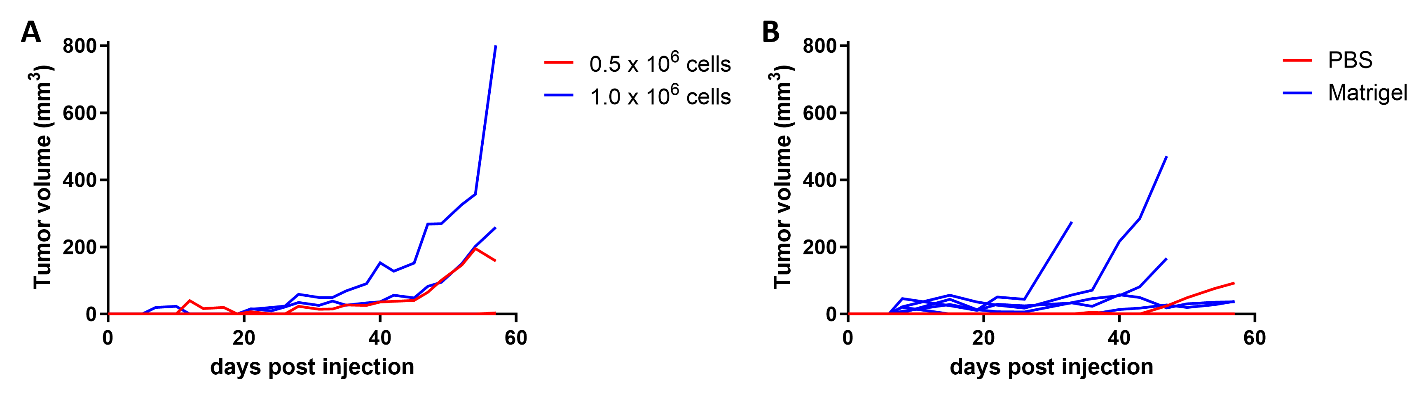


**Supplementary figure S1.** MOC1 parental tumor growth curves. (A) Tumor cells injected at the right hindlimb (0.5 x 10^6^ cells or 1.0 x 10^6^ cells in PBS), n = 3 per group. (B) Tumor cells injected at the right hindlimb (0.5 x 10^6^ cells in PBS or in matrigel), n = 5 per group. Curves represent individual mice.


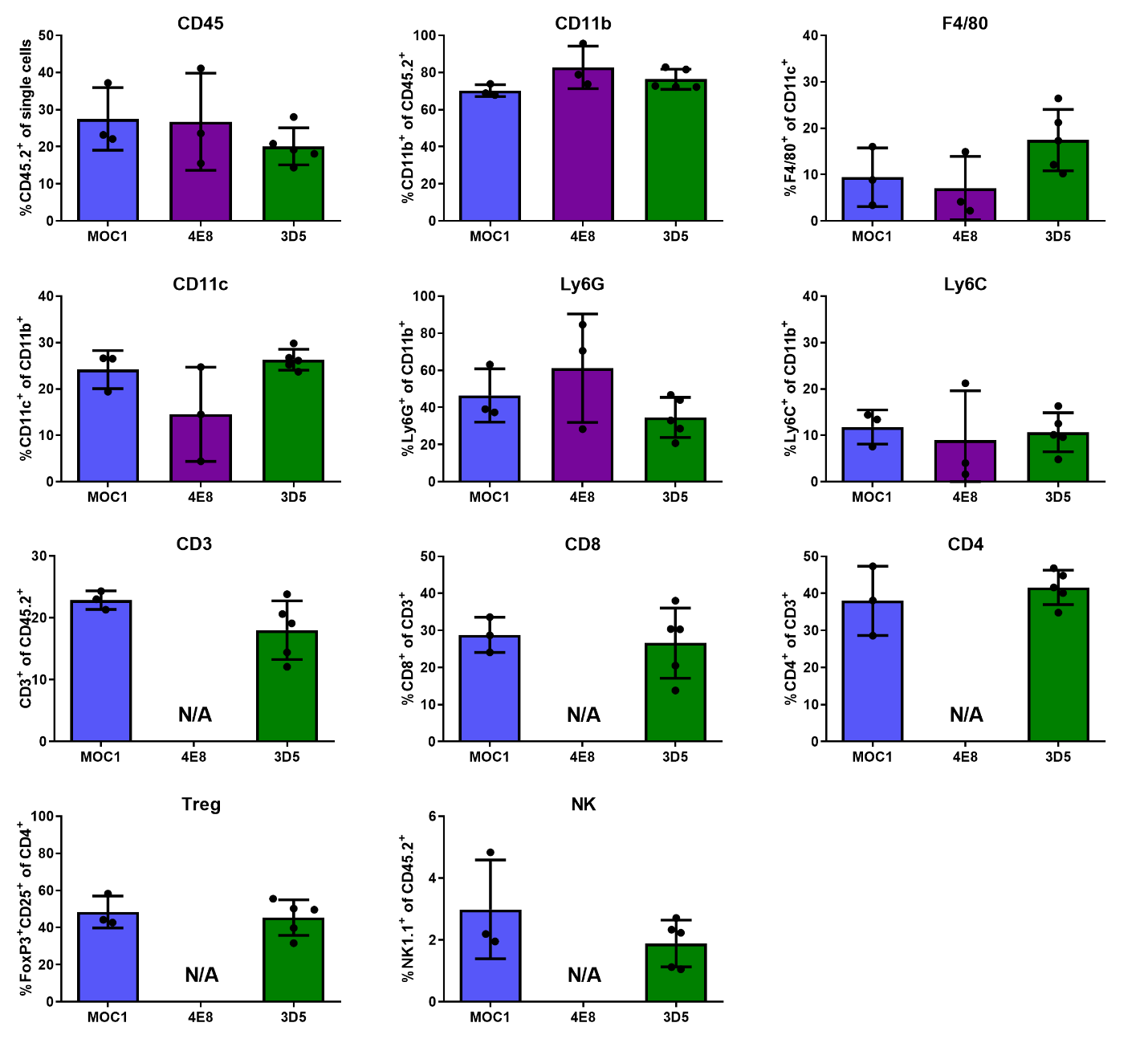


**Supplementary figure S2.** Presence of several immune cell subsets in the TME of MOC1 parental, MOC1.3D5^low^ and MOC1.4E8^intermediate^. N/A = data not available because of limited data points (N = 1). All graph bars show mean ± SD.


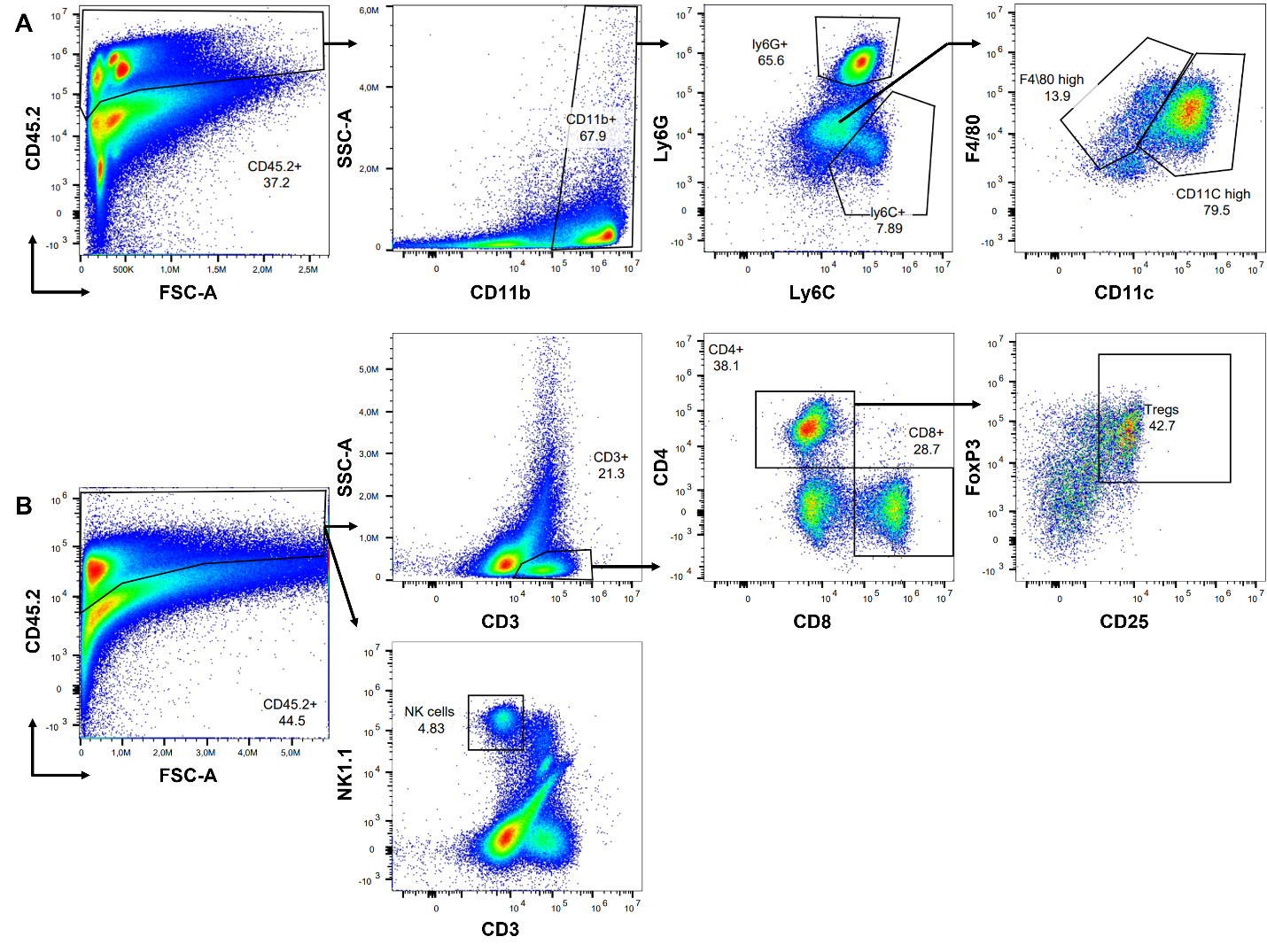


**Supplementary figure S3.** Gating strategy of live single cells for supplementary figure S2. (A) Gating strategy for myeloid cell subsets. (B) Gating strategy for T-cell subsets and NK cells.


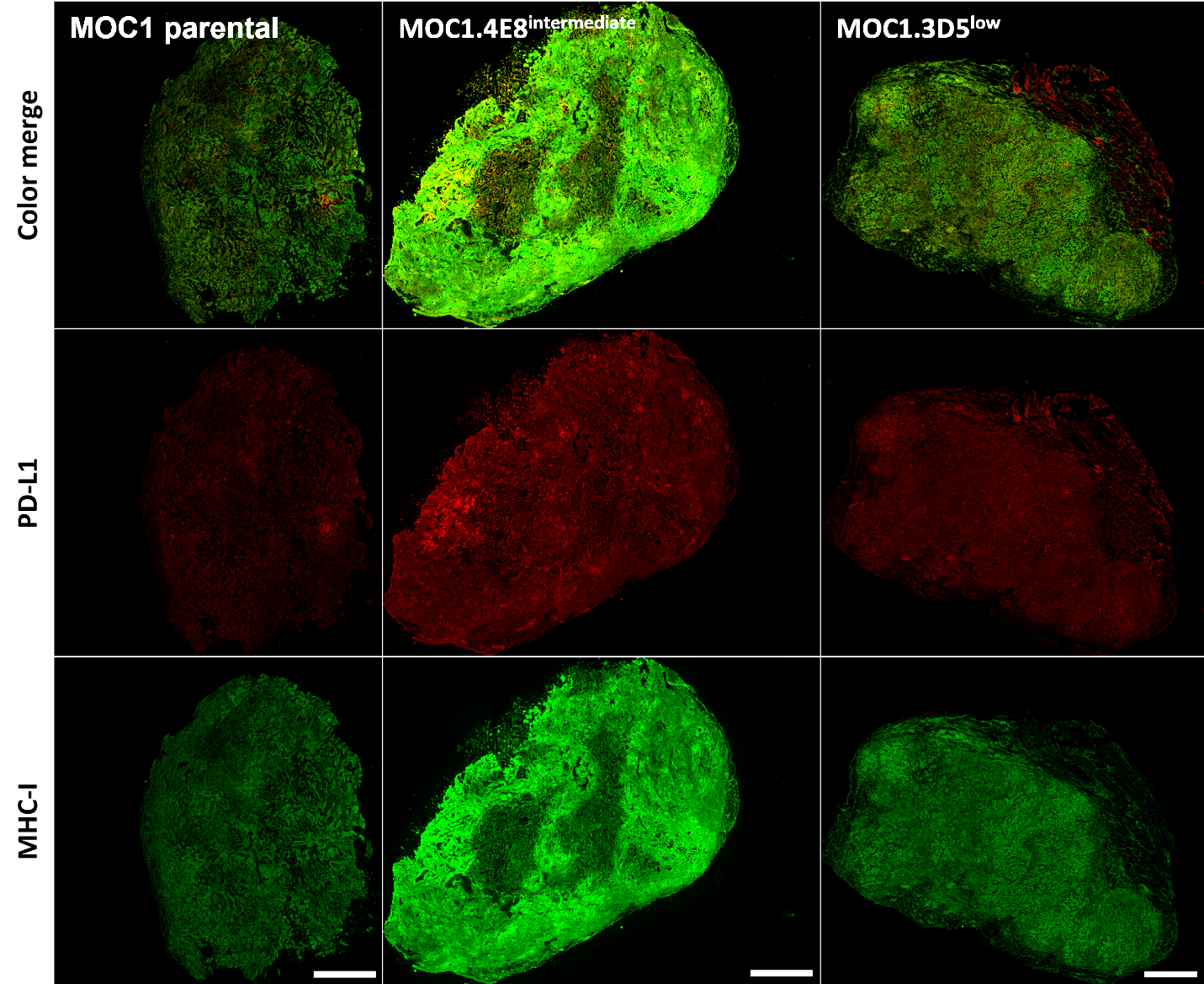


**Supplementary figure S4.** Color merge, PD-L1 and MHC-I images of immunohistochemical staining of PD-L1 (CD274, *red*) and MHC-I (H-2K^b^/H-2D^b^, *green*) on MOC1 parental, MOC1.4E8^intermediate^ and MOC1.3D5^low^ tumor sections. Scalebar represents 1.0 mm


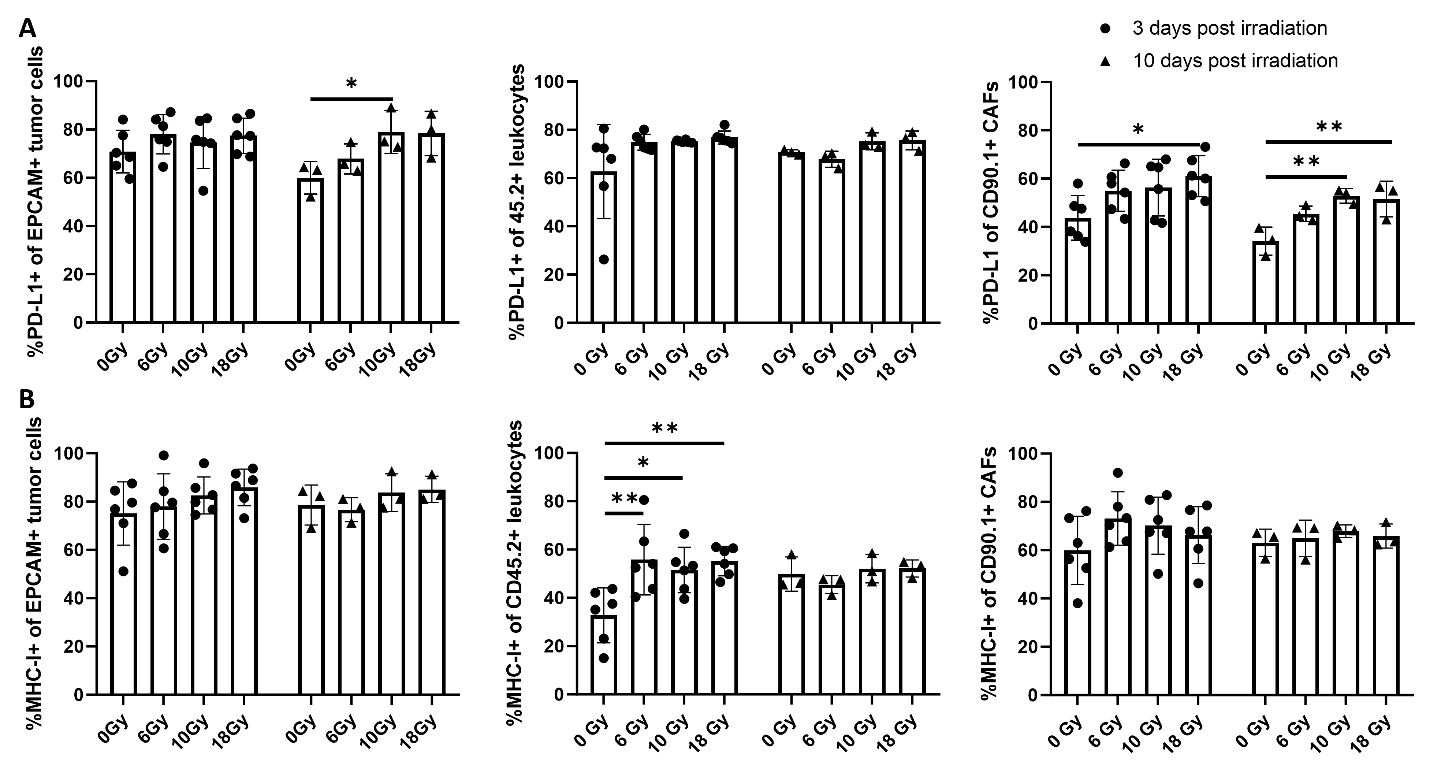


**Supplementary figure S5.** (A) % of PD-L1 and (B) MHC-I positive cells in MOC1.3D5^low^ derived CD45.2^-^/EpCAM^+^ tumor cells, CD45.2^+^ leukocytes and CD45.2^-^/EpCAM^-^/CD90.1^+^ CAFs. Graph bars show mean ± SD.


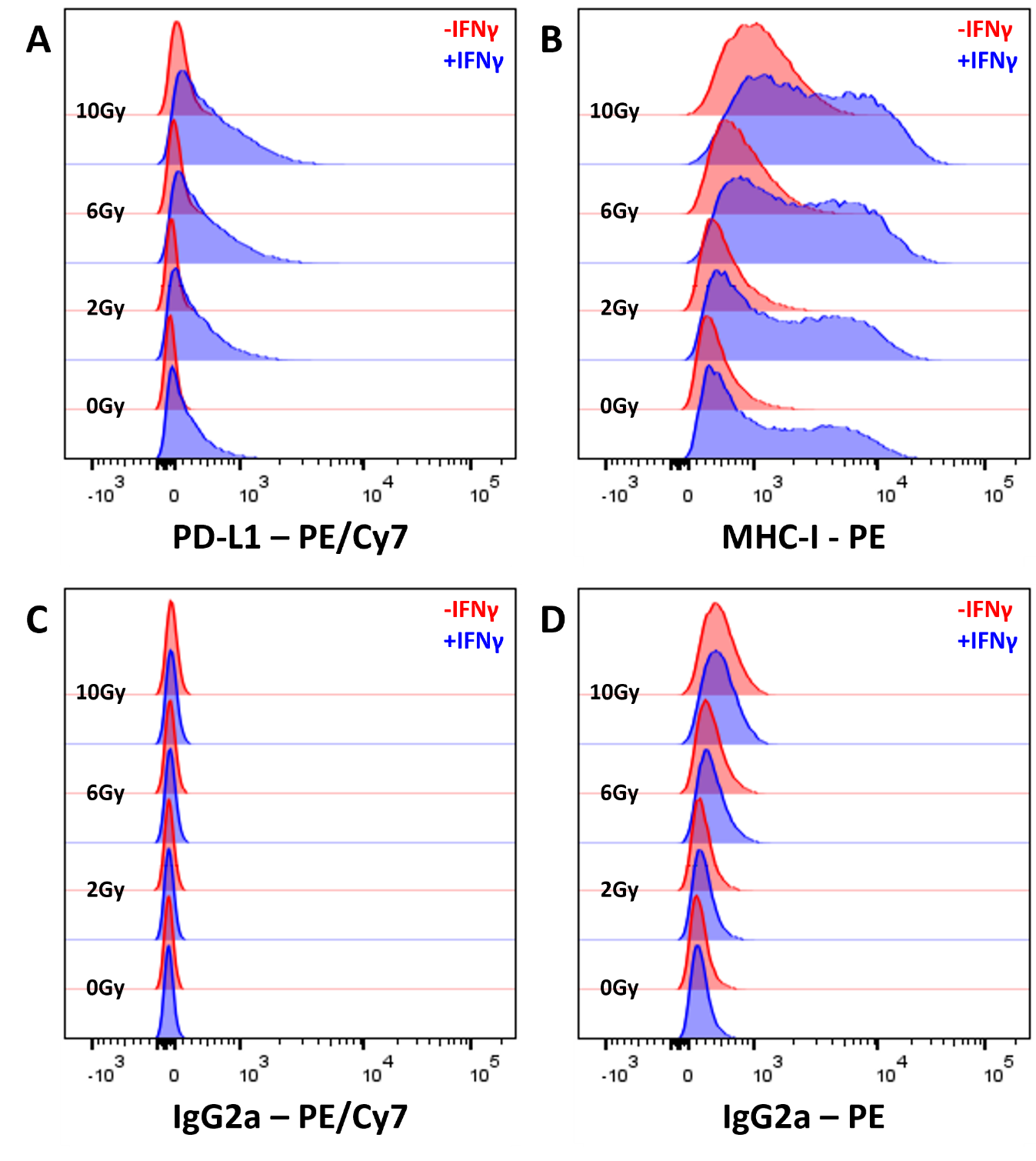
**Supplementary figure S6.** (A) PD-L1 expression and (B) MHC-I expression on MOC1.3D5^low^ cells *in vitro* and appropriate isotype controls (C-D) 24 h post irradiation (0, 2, 6 and 10 Gy) and/or 24 h treatment with IFNγ (5 ng/mL).


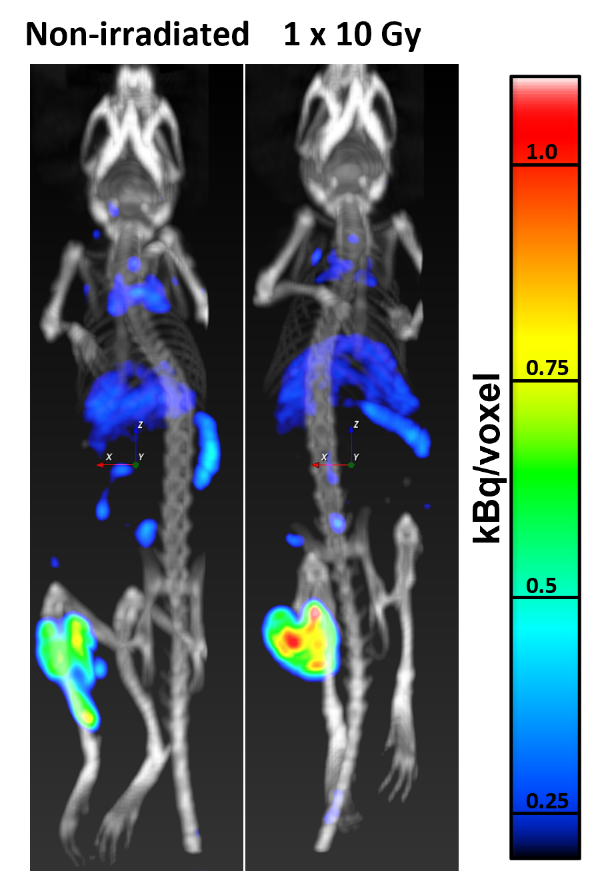


**Supplementary figure S7.** Coronal view of maximum intensity projections of [^111^In]In-anti-mPD-L1 microSPECT/CT in mice with non-irradiated and irradiated (1 x 10 Gy) tumors provided in figure 4A. Scale indicating the uptake in kBq/voxel.


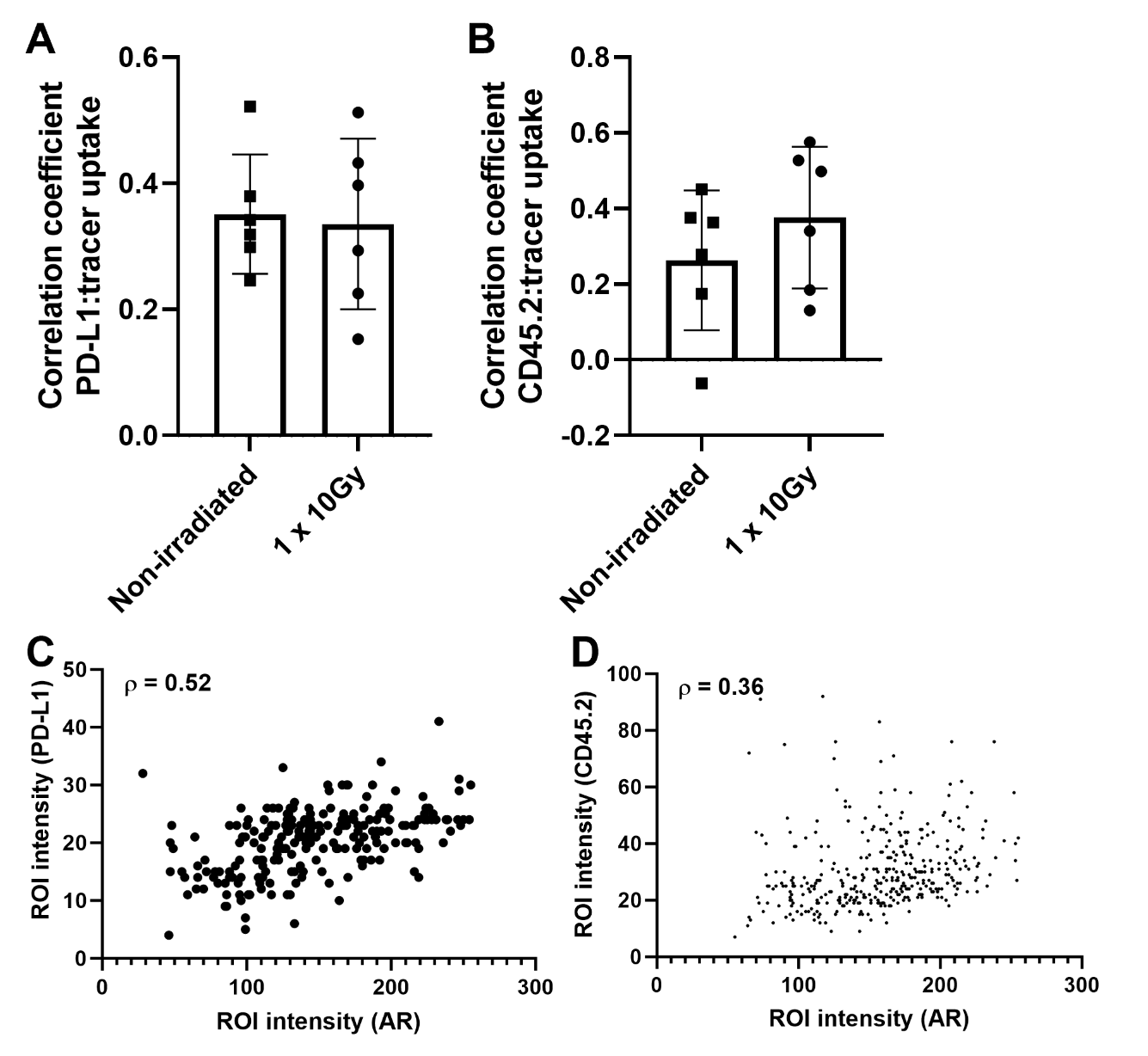


**Supplementary figure S8.** [^111^In]In-anti-mPD-L1 tracer uptake is correlated with PD-L1 expression and immune cell presence. (A) Spatial correlation between PD-L1 expression and tracer uptake as determined by co-registration and quantitative analysis of IHC and AR. Data points represent ρ of single mice. (B) Spatial correlation between CD45.2 expression and tracer uptake as determined by co-registration and quantitative analysis of IHC and AR. Data point represent ρ of single mice. (C-D) Examples of spatial correlation analysis of single mice showing region of interest (ROI) intensity (10 x 10 pixels) on (C) PD-L1 or (D) CD45.2 immunohistochemistry versus autoradiography. ρ = correlation coefficient.


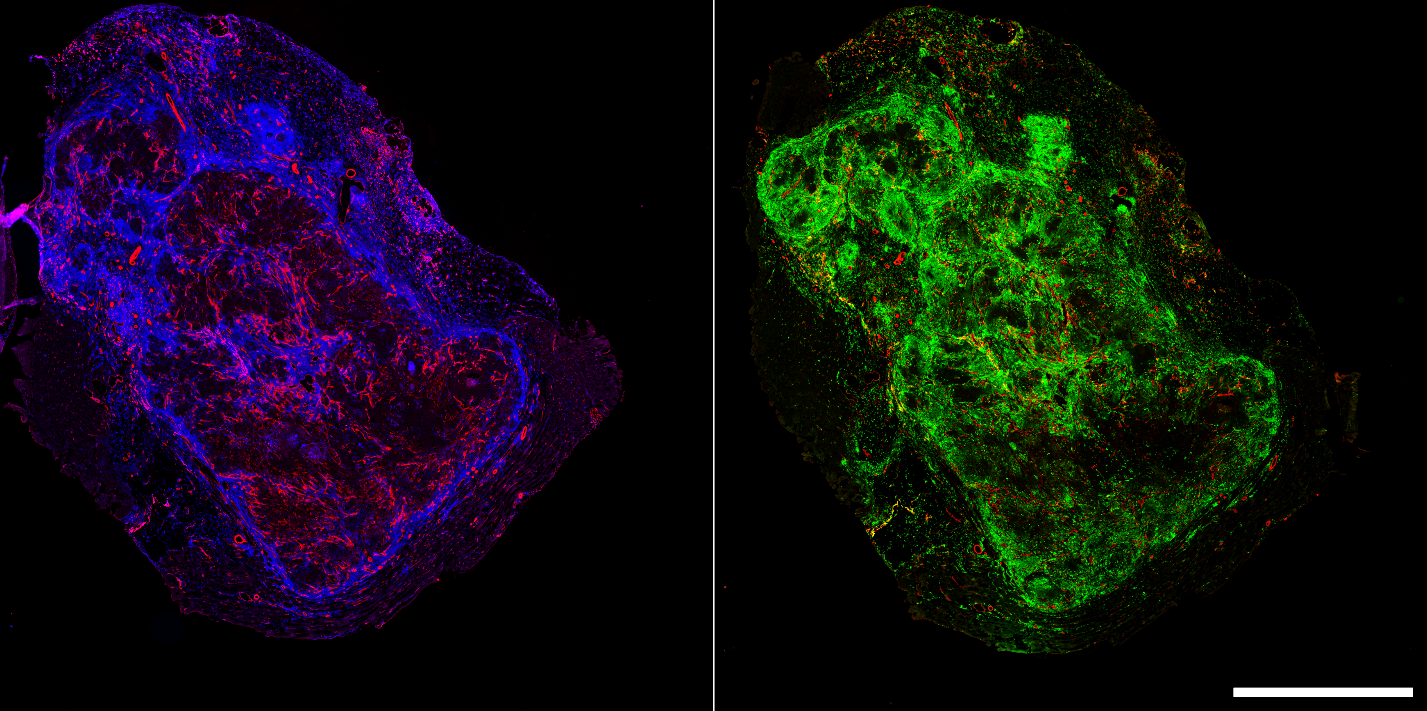


**Supplementary figure S9.** Color merge images of immunohistochemical staining of leukocytes (CD45.2, *blue*) and vessels (9F1, *red*) or MHC-II (I-A/I-E, *green*) and vessels on consecutive MOC1.3D5^low^ tumor sections. Scalebar represents 2.0 mm.

| **Tissue** | **[^111^In]In-anti-mPD-L1**  **Non-irradiated**  **(%ID/g)** | **[^111^In]In-anti-mPD-L1**  **1 x 10 Gy**  **(%ID/g)** | **[^111^In]In-IgG2b**  **Non-irradiated**  **(%ID/g)** | **[^111^In]In-IgG2b**  **1 x 10 Gy**  **(%ID/g)** |
| --- | --- | --- | --- | --- |
| **Blood** | 6.12 ± 0.96 | 6.37 ± 1.64 | 20.72 ± 1.82 | 19.14 ± 2.24 |
| **Muscle** | 1.33 ± 0.49 | 1.18 ± 0.44 | 1.53 ± 0.12 | 1.57 ± 0.19 |
| **Lung** | 8.66 ± 1.13 | 8.70 ± 0.84 | 7.69 ± 1.26 | 7.2 ± 1.00 |
| **Heart** | 4.35 ± 0.34 | 4.31 ± 0.34 | 5.68 ± 1.06 | 5.65 ± 0.97 |
| **Pancreas** | 1.78 ± 0.30 | 1.88 ± 0.25 | 2.98 ± 1.08 | 2.64 ± 0.69 |
| **Kidney** | 7.84 ± 0.49 | 7.73 ± 0.49 | 7.60 ± 0.82 | 7.50 ± 0.91 |
| **Liver** | 13.30 ± 2.50 | 12.50 ± 1.85 | 9.42 ± 1.45 | 7.63 ± 0.52 |
| **Stomach** | 2.83 ± 0.18 | 2.71 ± 0.27 | 2.59 ± 0.50 | 2.81 ± 0.57 |
| **Colon** | 3.22 ± 0.22 | 3.69 ± 0.49 | 2.65 ± 1.35 | 2.40 ± 0.33 |
| **Bone marrow** | 7.42 ± 0.62 | 7.92 ± 0.95 | 9.61 ± 1.44 | 12.98 ± 3.01 |
| **Bone** | 2.44 ± 0.22 | 2.32 ± 0.24 | 2.88 ± 0.65 | 4.06 ± 0.78 |
| **Thymus** | 5.73 ± 0.71 | 6.93 ± 0.98 | 4.05 ± 0.67 | 6.30 ± 0.99 |
| **Spleen** | 27.35 ± 2.06 | 25.24 ± 1.35 | 9.42 ± 0.98 | 10.26 ± 1.72 |
| **Duodenum** | 12.49 ± 3.49 | 10.59 ± 1.85 | 3.55 ± 0.72 | 4.16 ± 0.87 |
| **Lymph node draining** | 21.47 ± 3.61 | 22.10 ± 9.59 | 8.63 ± 1.72 | 11.65 ± 2.21 |
| **Lymph node contra lateral** | 13.72 ± 2.09 | 13.13 ± 4.86 | 10.54 ± 4.37 | 8.96 ± 1.23 |
| **Brown fat** | 17.7 ± 1.80 | 16.12 ± 1.28 | 5.39 ± 1.07 | 4.36 ± 0.32 |
| **Tumor** | 13.76 ± 2.05 | 16.83 ± 2.26 | 21.13 ± 4.17 | 20.93 ± 3.88 |
| **Tumor-to-blood ratio** | 2.75 ± 0.61 | 2.30 ± 0.49 | 1.09 ± 0.14 | 1.02 ± 0.16 |

**Supplementary table S1.** *Ex vivo* biodistribution of [^111^In]In-anti-mPD-L1 (30 µg, 24 h p.i.) and [^111^In]In-IgG2b (30 µg, 24 h p.i.) in C57BL/6 mice with subcutaneous MOC1.3D5^low^ tumor.
